# Influence of repeated sprint exercise on leukocyte morphology in adolescent athletes at different biological maturation rates

**DOI:** 10.3389/fphys.2025.1480776

**Published:** 2025-03-05

**Authors:** Fernanda Oliveira, Paulo Almeida-Neto, Geraldo Barroso Cavalcanti Júnior, Marcela Abbott Galvão Ururahy, Maria Angela Dantas, Breno Guilherme De Araujo Tinoco Cabral, Paulo Moreira Silva Dantas

**Affiliations:** ^1^ Federal University of Rio Grande do Norte, Natal, Brazil; ^2^ Blood Center Dalton Cunha - Hemonorte, Natal, Rio Grande do Norte, Brazil; ^3^ Health Sciences Center, Federal University of Rio Grande do Norte, Natal, Rio Grande do Norte, Brazil

**Keywords:** immunology, lymphocyte, children, sport, biological maturation

## Abstract

**Background:**

After performing strenuous physical exercises such as repeated sprint exercise (RSE), the leukocyte morphology undergoes changes suggesting immunodepression. Furthermore, it has been previously suggested that, in pediatrics, leukocyte changes may be influenced by the rates of the biological maturation (BM) process, which varies among individuals of similar chronological age.

**Objective:**

To investigate the influence of RSE on leukocyte morphology in adolescent athletes at different rates of BM.

**Methods:**

We conducted an experimental trial with a final sample consisting 21 adolescent athletes (male sex, age = 12.7 ± 1.2) underwent an RSE protocol, and blood samples were collected at “before, after, and 2 h post” moments. Based on the BM rates assessed by a predictive equation of skeletal age, participants were divided into two groups (Accelerated [n = 10] and Synchronized [n = 11]). Leukocyte morphology was analyzed microscopically and by leukogram.

**Results:**

Regardless of the BM rates, a time effect was observed on the absolute levels of total leukocytes (g/L: η^2^p = 0.36), lymphocytes (g/L: η^2^p = 0.50, %: η^2^p = 0.29), segmented neutrophils (g/L: η^2^p = 0.16, %: η^2^p = 0.43), and neutrophil-to-lymphocyte ratio [NLR] (g/L: η^2^p = 0.30). The synchronized BM group showed higher values than the accelerated group for total leukocyte levels (moments after and 2 h post [η^2^p = 0.10; p < 0.001]) and segmented neutrophils (moments after and 2 h post [η^2^p = 0.10; p < 0.001]).

**Conclusion:**

In adolescent athletes, changes caused by RSE in leukocyte morphology appear to be dependent on the BM rates.

## Graphical Abstract

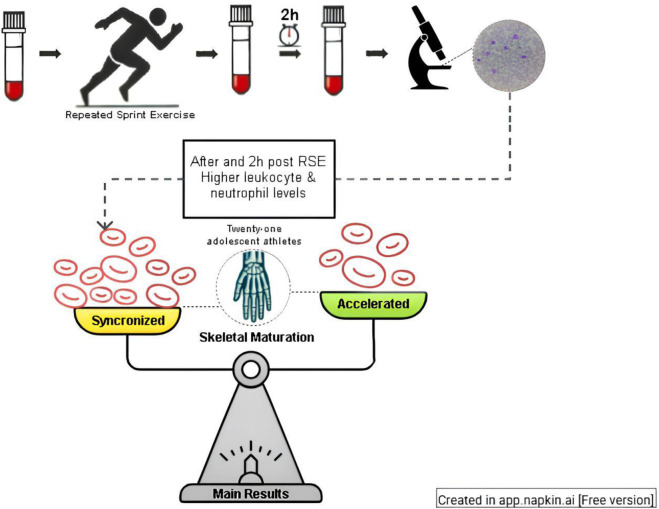

## Introduction

Repeated sprint exercise (RSE) has immunosuppressive potential (Akkoç et al., 2021), as it is an exhausting activity performed at supra-maximal intensity (“all-out”). Strenuous physical exercises promote significant changes in leukocyte morphology in adult athletes of different cardiorespiratory conditioning levels (Almeida-Neto et al., 2023a), which may promote the weakening of the immune system for a period that can range from two to 72 h after exercise (Shephard, 2010; Nieman, 2000). Recently, a review suggested that, in adolescent athletes, immune responses resulting from strenuous physical exercise may depend on biological maturation (BM), which refers to the enhancement of human biological systems, including the lymphatic system (Almeida-Neto et al., 2023b).

BM can be assessed by somatic markers that focus on tissue development (e.g., peak height velocity and predicted adult height), puberty markers (e.g., hormone levels, pubic hair, and genital size), and the rate of skeletal calcification (e.g., skeletal age and dental eruption) (Almeida-Neto et al., 2023b; de Almeida‐Neto et al., 2022). Among the BM markers mentioned, the maturity rate assessed by skeletal age is considered the gold standard (de Almeida‐Neto et al., 2022; Cabral et al., 2013), enabling the determination of whether the development of biological systems in children and adolescents is occurring at a delayed, synchronized, or accelerated rate compared to their chronological age (Malina and Bouchard, 2002). The morphology of leukocytes, in turn, can be analyzed using optical microscopy through a method commonly known as “differential count,” which allows for the differentiation of the structure of leukocyte subpopulations.

Thus, the initial hypothesis of this study is that, after performing RSE, adolescent athletes with an “accelerated” BM rate will suffer less impact from RSE on leukocyte morphology due to their bodies being biologically more advanced compared to their peers with a slower BM rate. Therefore, the objective of this study was to verify the influence of RSE on the leukocyte morphology of adolescent athletes at different BM rates, assessed by skeletal age.

## Methods

**FIGURE 1:**
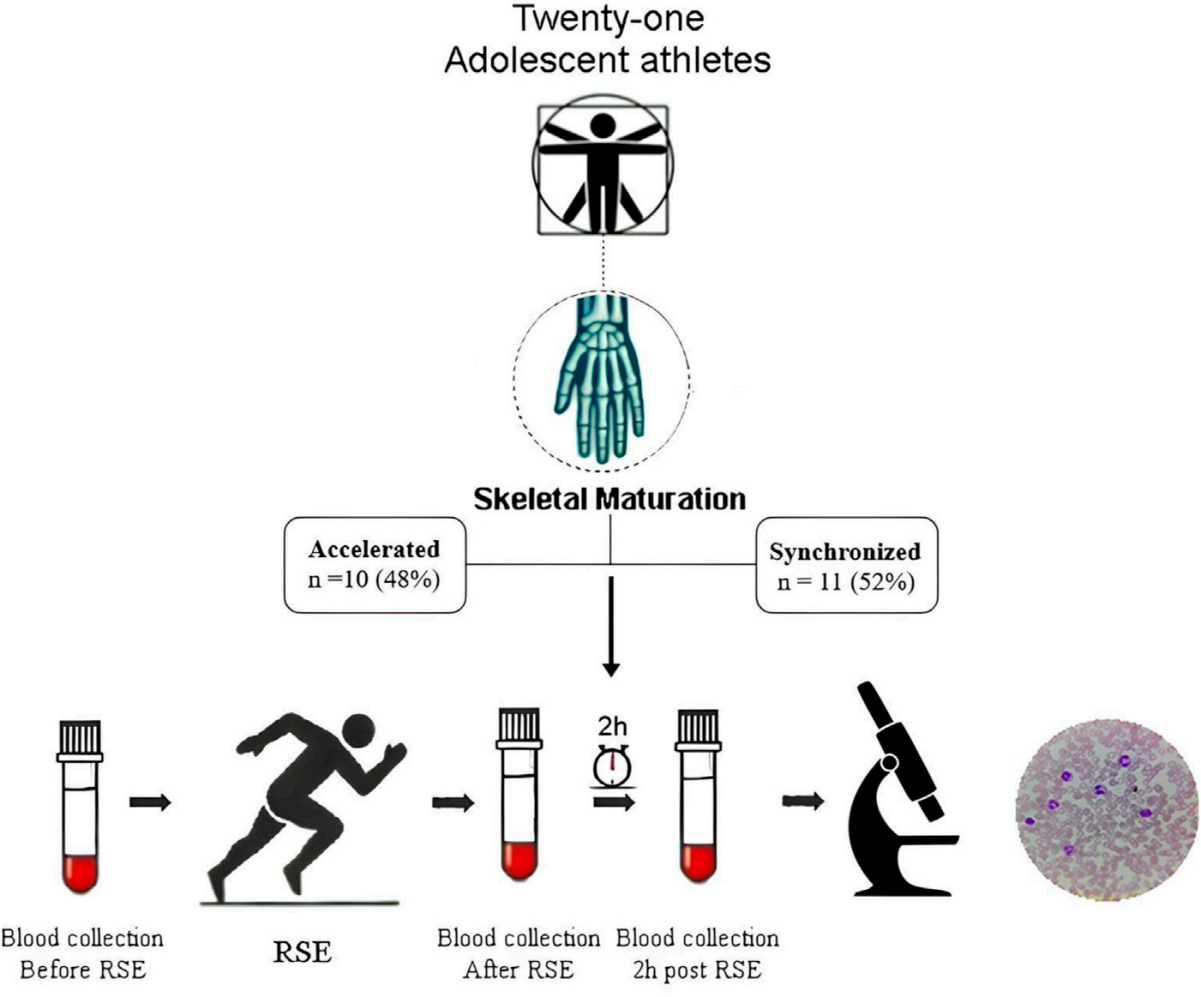
Procedures. RSE: Repeated sprint exercise. This figure was created in app. napkin.ai [free version].

We conducted an experimental trial with a final sample consisting of 21 male adolescent athletes [Age: 12.7 ± 1.2 years. Modalities: Jiu-jitsu (n = 13), soccer (n = 7), volleyball (n = 1)]. Eight were regional-level competitors, two were national-level competitors, and one was an international-level competitor. The competitive level was classified based on the criteria outlined by McKay et al. (2021). The participants were divided according to skeletal maturation stage (10 in the accelerated stage and 11 in the synchronized stage) (Figure 1).

Participants were recruited from sports academies and clubs in the city of Natal, RN. The inclusion criteria were: (i) being involved in a combat or court sport and participating in regional, national, or international level competitions for at least 1 year before the study; (ii) maintaining a minimum training frequency of two weekly sessions, lasting at least 1 hour per session, in the 6 months preceding the study; (iii) having a high level of physical activity, equal to or greater than 3,000 METs weekly. The exclusion criteria were defined as: (i) exhibiting symptoms of upper respiratory tract infection at the time of recruitment; (ii) use supplements that could interact with the immune system (e.g., glutamine, vitamin C, omega-3, *etc.*) or any immunomodulatory medications (e.g., baricitinib, tocilizumab, corticosteroids, *etc.*); (iii) have any clinically diagnosed disease; or (v) have suffered a musculoskeletal injury within the 6 months prior to the study.

## Sample size

The sample size was calculated *a priori* based on a pilot study composed of 10 athletes (5 in an accelerated maturation stage and 5 in a synchronized maturation stage). Thus, we analyzed total leukocyte levels at pre, immediately, and 2 h post-RSE. Subsequently, we identified an effect size (η^2^p) of 0.5 (time effect). Therefore, using the G*Power^®^ software (Version 3.1, Düsseldorf, Germany), we considered an α of 5% and a β of 0.8, arriving at a minimum sample size of eight subjects per group (Power: 0.82). For possible sample losses, we aimed to add between 25% and 35% more than the minimum sample size per group. We highlight that in the present study there was no sample loss.

## Ethics

This study was submitted to the Ethics Committee of the Federal University of Rio Grande do Norte, Brazil, and obtained approval (#76447923.3.0000.5537), in compliance with the Declaration of Helsinki. The study design was publicly available on the Open Science Framework Registries platform (DOI: 10.17605/OSF.IO/HF9V3). All participants and their respective guardians were duly informed about the study procedures. Those who agreed to participate had their guardians sign the consent form and the adolescents sign the assent form, demonstrating their free and informed consent to participate in the study.

## Procedures

The participants’ anthropometric data were assessed the day before the experiment. On the day of the intervention, they arrived at the data collection site at 7 a.m. and were directed to a reserved room for the first blood draw (pre-exercise). Afterward, they performed a 5-minute warm-up, consisting of jumps and short walks on the athletics track, in preparation for the RSE protocol. Immediately after completing the exercise session, a second blood sample was collected in a tent set up at the edge of the track. The athletes were then taken to a climate-controlled room at 24°C, where they rested for 2 hours until the third blood collection (Figure 1). Following these procedures, blood smears were prepared on glass slides for leukocyte morphology analysis using optical microscopy.

## Blinding

During data collection, adolescents was unaware of their BM stage, nor were researchers aware. A collaborator external to the research applied the intervention, ensuring neutrality during this stage. During the analysis of leukocyte morphology using a microscope, the data were masked, meaning that the evaluators did not know which participant or group the glass slide belonged to. Finally, statistical analyses were carried out by a collaborator external to the research.

## Physical activity level

The level of physical activity was assessed using the web version of the International Physical Activity Questionnaire, which is validated to analyze metabolic equivalents (METs) related to energy expenditure in physical activities (Pires et al., 2014). The questionnaire covers activities at work, transportation, domestic activities, and leisure/sport activities. The web-IPAQ automatically calculates METs for habitual energy expenditure, providing scores for each category and a total score. Based on the total METs, the web-IPAQ categorizes the level of physical activity as low, moderate, or high (Ainsworth et al., 1993). The web-IPAQ was self-reported, for this purpose the participants completed the web-IPAQ in the presence of at least one person responsible to assist in reporting the activities carried out and a researcher to assist in interpreting the questions. All participants in this study reported a high level of physical activity according to the web-IPAQ classification, thus meeting the required inclusion criterion.

## Upper respiratory tract infection symptoms

In the week preceding the study during the recruitment phase, we assessed upper respiratory tract infection symptoms (URTIS) using the Wisconsin Upper Respiratory Symptom Survey-21 (WURSS-21) (Barrett et al., 2002; Barrett et al., 2009). Thus, only volunteers who did not report URTIS remained in the study. Notably, the tool was previously used in a study analyzing young athletes (Brunelli et al., 2014) and was validated for pediatric populations (Schmit et al., 2021). We reapplied the WURSS-21 on the day of the RSE intervention, and URTIS did not change compared to the evaluation performed during the recruitment phase.

## Anthropometry

Participants, barefoot and wearing light clothing, had their body mass measured using a digital Filizola^®^ scale with a capacity of 200 kg and an accuracy of 0.10 kg (São Paulo, Brazil). Height was determined using a Sanny^®^ stadiometer (accuracy of 0.1 mm) (São Paulo, Brazil). The humerus and femur bone diameters were measured with a Sanny^®^ analog caliper (accuracy of 0.1 mm). The triceps skinfold was analyzed with a Sanny^®^ analog scientific caliper (accuracy of 0.1 mm). Arm circumference was measured with a Sanny^®^ steel anthropometric tape (accuracy of 0.1 cm). All measurements were taken by a single evaluator following the protocols of the International Society for the Advancement of Kinanthropometry (ISAK) (Silva et al., 2020). The intra-observer technical error of anthropometric measurements was ≤1.0% (Adao Perini et al., 2005). Fat and lean mass levels were analyzed by dual-energy X-ray absorptiometry (DXA) using a LUNAR^®^/GE PRODIGY - LNR 41,990 device (Washington, DC, United States). The software used was enCORE, GE Healthcare^®^, version 15.0 (Madison, WI, United States). Appropriate algorithms for the pediatric population were employed (Khadilkar et al., 2020). During the evaluations, the DXA equipment operated with the following configuration: whole-body assessment, voltage (kV): 76.0, current (mA): 0.150, radiation dose (µGy): 0.4 (very low, without health risk). Basal metabolic rate was estimated using automated algorithms through DXA equipment analyses, based on body composition results.

## Dietary pattern

During the procedures of the present study, participants were instructed to restrict the consumption of stimulating foods (e.g., caffeine, taurine, pepper, *etc.*). Additionally, the dietary pattern was assessed using 24-hour dietary recall questionnaires (R-24 h) (Gibson, 2005; Castell et al., 2015). The first questionnaire was administered 24 h before the intervention (atypical day), and the second on the day of the intervention (typical day). It is important to note that the R-24 h questionnaires from all days were examined by nutrition professionals who, according to the analyses conducted, found that the participants’ dietary patterns did not change throughout the study.

## Sleep pattern

The sleep pattern was analyzed using a sleep diary (Reed and Sacco, 2016). Participants recorded the time they went to bed, their perception of the time spent in bed until they fell asleep, the time they woke up, the total time in bed, and if they woke up during the night. Based on these data, we calculated sleep efficiency. During the study period, all participants had sleep efficiency between 90.4% and 99.8% 24 h before the RSE and between 85.4% and 99.5% on the day of the RSE, indicating good sleep efficiency (Reed and Sacco, 2016).

## Biological maturation

BM was determined using the following mathematical model for estimating skeletal age [for boys and girls aged eight to 14 years, in both sex, the equation explains 75.4% of the skeletal age variation measured by X-ray (*r* ^2^ = 0.754, standard error = 1.243)] (Cabral et al., 2013):

Skeletal age years=−11.620+7.004×Height m+1.226×Dsex+0.749×Age years−0.068×Triceps skinfold mm+0.214×Corrected arm circumference cm−0.588×Humerus diameter cm+0.388×Femoral diameter cm

Dsex: For male sex = 0; for female sex = 1. (m): meters. (mm): millimeters. (cm): centimeters.

Corrected arm circumference was determined by the equation:

Corrected Arm Circumference cm=Contracted biceps circumference cm−Triceps skinfold mm/10

(mm): millimeters. (cm): centimeters.

After determining skeletal age, to classify BM rate:

Skeletal age−Chronological age.

Scores generated from this classify skeletal maturation rate as: delayed (<−1.0), synchronized (between −1.0 and 1.0), and accelerated (>1.0).

## Repeated sprint exercise (RSE)

In the 24 h preceding the RSE, participants were instructed not to engage in strenuous physical activity. The RSE consisted of 18 high-intensity sprints performed on an official athletic track, with an average temperature of 26.2°C ± 1.5°C and an average wind speed of 1.2 ± 0.3 m/s. The RSE comprised three sets of six 35-meter sprints, with sets interspersed with 5 min of passive rest and sprints with 10 s of passive rest. Notably, the mean time for the worst sprint was 6.9 s and for the best sprint was 5.9 s.

## Monitoring subjective effort perception

We used the Rating of Perceived Exertion (RPE) scale to analyze RSE intensity, monitoring RPE according to Borg’s scale (Borg, 1982). Scale exposure occurred after the last sprint of the RSE. The monochromatic visual scale ranged numerically from six to 20, where six indicated absolute rest and 20 indicated maximum effort. It is important to note that participants were familiarized with the RPE 24 h before the RSE. The average RPE for this study was 17.5, indicating that participants perceived the RSE as highly intense.

## Blood collection and hematological analysis

Blood was collected from the antecubital vein of participants’ upper limbs using a vacuum collection system. At each collection (pre, immediately post, and 2 h post-RSE), 10 mL of peripheral blood (PB) was drawn into tubes containing EDTA anticoagulant (BD-Vacutainer, EDTA-K2 5.4 mg Plus Plastic). White blood cell (WBC) count was conducted using automated cell counting (Cell-Dyn 3,000, Unipath Corp., Mountain View, CA). Peripheral blood smears were prepared on neutral glass slides (with 45° beveled corners, Size: 25.4 × 76.2 ± 0.5 mm, Thickness: 1.0–1.2 mm), air-dried, and stained with May-Grunwald Giemsa (MGG, Laborclin, Brazil). Subsequently, for leukocyte morphology analysis, hematologic cells (lymphocytes, monocytes, segmented neutrophils, and rod neutrophils) were counted under an optical microscope using 100x objective lenses (Zeiss^®^ microscope, Gütting, Germany). To convert to absolute values, percentage values were multiplied by the absolute number of leukocytes and divided by 100. It is noteworthy that hematologic cell counting was performed by two researchers with extensive experience, achieving high agreement (ICC = 0.93). Evaluators used the WBC Counter smartphone app (K.Sasa^®^, version 3.1, United States) to record observed cells. The mean count determined by evaluators was considered. To determine systemic inflammation levels, we calculated the total neutrophil-to-lymphocyte ratio.

## Statistics

Data normality was tested using Shapiro-Wilk tests and QQ-plotting. Data were expressed as mean and standard deviation (±), with minimum and maximum values. Independent sample t-tests were used for comparisons between groups (accelerated vs synchronized) at baseline. For analyses considering time (pre, immediate, and 2 h post-RSE) and condition (BM paces: synchronized and accelerated), a general linear model (GLM) was used (Kim and Timm, 2006; Gallucci, 2019). Sub-analyses by sport type (combat sports and court sports) were also conducted using GLM. Bonferroni *post hoc* tests were used to verify specific differences. Effect size between differences and GLM model effect were assessed using partial eta-squared (η^2^p), interpreted by magnitude (Cohen, 1988): small (<0.01), medium (between 0.02 and 0.06) and large (>0.14). All analyses were performed using open-source software Jamovi^®^ (version: 2.3.18. Sydney, Australia), with significance set at p < 0.05. And addition through the Eta values to calculate the power of the analyses performed, for this we used the G*Power^®^ software (Version 3.1, Düsseldorf, Germany). All figures and graphical analyses were performed in GraphPad Prism software (Version 8.01 (244), California, United States).

## Results

According to data characterization (Table 1), the “accelerated” maturation stage group showed higher lean mass levels compared to the “synchronized” maturation stage group (p = 0.01). For other characterization variables, no statistically significant differences were observed (p > 0.05). This suggests that the level of sports training was equivalent between the analyzed groups.

**TABLE 1:** Sample characterization.

Variable	Total (n: 21)		Accelerated (n:10)		Synchronized (n: 11)	
	Average ±	Minimum; Maximum	Average ±	Minimum; Maximum	Average ±	Minimum; Maximum
Chronological age (years)	12.7 ± 1.2	10.8; 14.9	13.1 ± 1.4	11.0; 14.9	12.4 ± 0.9	10.8; 13.9
Skeletal age (years)	13.5 ± 2.0	10.6; 16.9	15.2 ± 1.5	11.7; 16.9	11.9 ± 0.8	10.6; 12.9
BM rate (Score)	0.9 ± 1.2	−0.9; 2.7	1.8 ± 0.5	1.0; 2.7	0.0 ± 0.7	−0.9; 0.9
Height (cm)	157.4 ± 11.8	138.0; 181.0	163.9 ± 12.7	138.0; 181.0	151.3 ± 7.2	141.2; 164.0
Mass (kg)	51.1 ± 15.7	32.8; 97.5	56.9 ± 19.3	33.4; 97.5	45.8 ± 9.8	32.8; 62.3
Fat mass (kg)	12.1 ± 8.2	4.4; 34.9	13.0 ± 9.9	4.4; 34.9	11.3 ± 6.5	5.1; 23.9
Lean mass (kg)	33.7 ± 10.5	21.0; 29.2	39.6 ± 11.5	21.4; 59.2	28.3 ± 5.9	21.0; 35.6
Basal metabolic rate (Kcal)	1.5 ± 0.3	1.2; 2.5	1.6 ± 0.4	1.2; 2.5	1.4 ± 0.2	1.2; 1.8
Time spent practicing sports (years)	5.0 ± 3.0	1.0; 10.0	5.8 ± 3.7	1.0; 10.0	4.3 ± 2.1	1.3; 7.0
Weekly training load (sessions)	4.7 ± 1.0	3.0; 7.0	5.0 ± 0.8	3.0; 6.0	4.5 ± 1.2	3.0; 7.0
Training load per session (hours)	1.4 ± 0.3	1.0; 2.0	1.4 ± 0.3	1.0; 2.0	1.3 ± 0.3	1.0; 2.0

Variable	Total (n: 21)		Accelerated (n:10)		Synchronized (n: 11)	
Data on sports practice	n	Percent	n	Percent	n	Percent
Fighting sport	13.0	62.0%	7.0	70.0%	6.0	54.0%
Court sport	8.0	38.0%	3.0	30.0%	5.0	46.0%

BM: biological maturation. ±: standard deviation. n: absolute number. %: percentage. cm: centimeters. Kg: kilograms. Kcal: calories.

**FIGURE 2:**
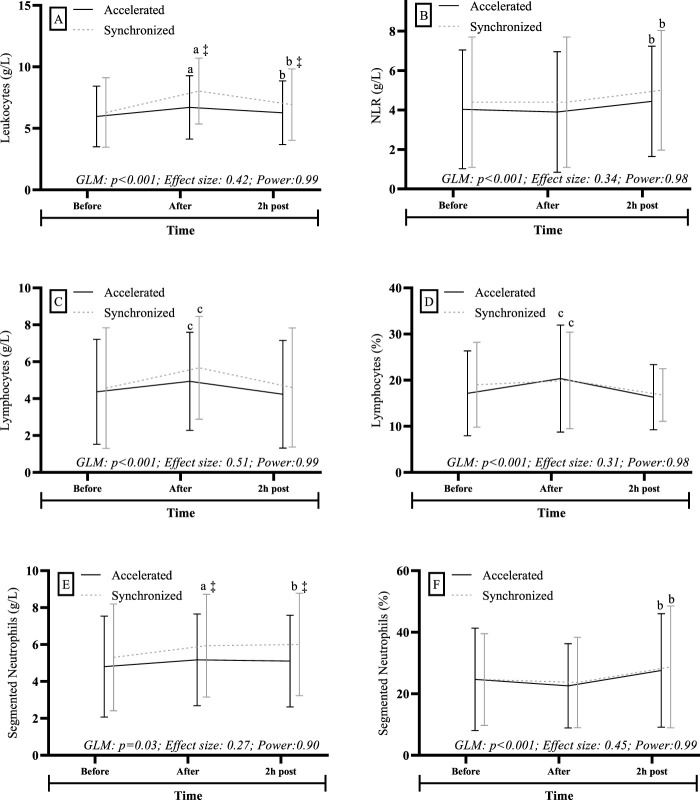
General linear model to make comparisons considering the different rates of skeletal maturation (synchronized and accelerated) and the effect of time (before, after and 2 h post). GLM: General Linear Model. NLR: Total neutrophils/lymphocytes ratio. g/L: Cell per liter. %: Percentage. ‡: Higher than the Accelerated group [Post hoc Bonferroni (p < 0.05)]. **(A)** Higher than the Before moment [Post hoc Bonferroni (p < 0.05)]. **(B)** Superior to the Before and After moments [Post hoc Bonferroni (p < 0.05)]. **(C)** Higher than Before and 2 h post moments [Post hoc Bonferroni (p < 0.05)]. **(A)**: analysis related to lymphocytes. **(B)**: analysis related to NLR. **(C,D)**: analysis related to lymphocytes. **(E,F)**: analysis related to segmented neutrophils.

In comparisons between different BM rates, subjects in the “synchronized” rate showed higher total leukocyte values compared to their counterparts in the “accelerated” rate during the “After” and “2 h post” RSE time points (Condition: p < 0.001; Effect size: 0.10 | Power: 0.90) (see Figures 2A). A similar result was observed for absolute levels of segmented neutrophils, where subjects in the “synchronized” rate showed higher values than their “accelerated” rate counterparts during the “After” and “2 h post” RSE time points (Condition: p = 0.03; Effect size: 0.10 | Power: 0.90) (Figure 2E).

Regardless of BM rate, a time effect was observed on absolute leukocyte levels showing a continued increase at each time point (the “After” time point was higher than “Before”, and “2 h post” was higher than “Before” and “After” time points) (Time: p < 0.001; Effect size: 0.36 | Power: 0.98) (see Figure 2A). Similarly, there were differences in absolute levels and percentage of lymphocytes (the “After” time point was higher than “Before” and “2 h post”) [Absolute levels: Time: p < 0.001; Effect size: 0.50 | Power: 0.99 (Figure 2C). Percentage: Time: p < 0.001; Effect size: 0.29 | Power: 0.98 (Figure 2D)]. Percentage of segmented neutrophils [Time: p < 0.001; Effect size: 0.43 | Power: 0.99 (Figure 2F)], and absolute levels of neutrophil-to-lymphocyte ratio (NLR) (the “2 h post” time point was higher than “Before” and “After” time points) (Time: p < 0.001; Effect size: 0.30 | Power: 0.98) (Figures 2B).

For subjects in the “synchronized” BM rate, a time effect on segmented neutrophils at “After” was observed, which was higher than “Before” (Interaction–Condition/Time: p < 0.001; Effect size: 0.11 | Power: 0.90) (see Figures 2E). No significant effects of condition or time were observed for monocyte levels (GLM: p > 0.4; effect size <0.07 | power <0.09), as well as for rod neutrophil levels (GLM: p > 0.08; effect size <0.05 | power <0.5). Further details regarding the results for monocytes and rod neutrophils can be found in *Supplementary Table S-1*.

## Sub-analyses

Sub-analyses (using a general linear model [GLM]) were conducted to assess whether the type of sport practiced (combat sports vs court sports) would affect the results of the current study. Thus, the type of sport did not show a significant effect on total leukocyte levels (η^2^p: <0.10, p > 0.05; Power: 0.90). Similarly, no differences were observed between types of sports practiced for the other analyzed variables (η^2^p: <0.13, p > 0.05; Power: 0.90).

## Discussion

This study aimed to investigate the influence of RSE on leukocyte morphology in adolescent athletes at different rates of BM analyzed by skeletal age. Our initial hypothesis was that subjects in an accelerated BM rate might experience less impact from RSE due to their biologically more mature organism compared to their peers in slower BM rate. The main results support this initial hypothesis. Additionally, sub-analyses revealed that the type of sport practiced (combat sports vs court sports) did not show a significant effect on the analyzed variables.

### Biological maturation and leukocyte morphology

According to the results of this study (Figure 2), individuals in synchronized BM rate exhibited higher levels of total leukocytes and segmented neutrophils at “After” and “2 h post” RSE compared to their counterparts in accelerated BM rate. A possible explanation is that those who mature faster have more developed biological systems (e.g., musculoskeletal, endocrine, immune, *etc.*) (Malina and Bouchard, 2002; Rowland, 2005), suggesting that these individuals have more enhanced lymphoid tissue than those maturing more slowly (Almeida-Neto et al., 2023b), implying that subjects in accelerated BM rate are more resistant to immune stress induced by RSE.

Adolescent athletes in advanced BM stages also demonstrate greater glycolytic efficiency, likely due to higher activity of anaerobic enzymes (Kaczor et al., 2005; de Almeida-Neto et al., 2022). This could suggest that the neuromuscular demands during RSE were relatively lower in the accelerated BM group, potentially influencing their leukocyte responses. The reduced physiological strain in this group might have contributed to their ability to maintain immune recovery more effectively.

During adolescence, the expansion of the thymus gland plays a crucial role in immune system development and efficiency (Almeida-Neto et al., 2023b; Scammon and Calkins, 1929). This expansion, more pronounced in biologically mature individuals (e.g., accelerated BM rate), may provide an immunological advantage (Almeida-Neto et al., 2023b). Such factors may also help explain the morphological differences in leukocytes observed in this study, reinforcing the importance of BM related variations in immune responses.

Beyond the nuances of BM processes, additional mechanisms may have contributed to the observed findings (Wang et al., 2020). For instance, catecholamines like adrenaline, released during exercise, bind to β-adrenergic receptors on leukocytes, mobilizing them into the bloodstream (Peake et al., 2017). Furthermore, exercise-induced shear stress facilitates the release of immune cells from vessel walls, particularly in secondary lymphoid tissues such as the lungs, spleen, and liver, allowing their entry into circulation (Simpson et al., 2015).

The increase in leukocyte counts during exercise is predominantly driven by neutrophils and lymphocytes (Wang et al., 2020; Simpson et al., 2015). While neutrophil levels tend to rise during recovery, lymphocyte counts typically decrease immediately after exercise (Wahl et al., 2020; Gonçalves et al., 2020). Baseline levels for both are generally restored within 24 h (Gonçalves et al., 2020; Souza et al., 2021). However, in the present study, within the first 2 hours of recovery, individuals with accelerated BM rates appeared to demonstrate an advantage in the recovery of total leukocyte and segmented neutrophil counts compared to their synchronized BM counterparts (Figure 2).

### Repeated sprint exercise and leukocyte morphology

This study identified that up to 2 h post RSE for synchronized BM rate group, systemic inflammation assessed by NLR was 50% higher compared to before RSE (Figure 2). Whereas in accelerated BM rate group, 2 h post RSE showed a 20% increase in NLR compared to before RSE. Although both groups experienced a time effect, when relativizing differences compared to before RSE, it is possible to suggest that subjects in accelerated BM rate were less susceptible to systemic inflammation promoted by RSE compared to their synchronized BM rate counterparts.

This can be justified by the fact that subjects in accelerated maturation rate show greater efficiency in the stretch-shortening cycle (i.e., the primary biomechanical mechanism for repeated sprints and jumping activities) compared to their slower BM rate counterparts (Dantas et al., 2020). Thus, there is less neuromuscular system wear and tear in these subjects, resulting in lower levels of systemic inflammation due to neuromuscular stress induced by physical exercise. It is known that strenuous exercise leads to significant damage to skeletal muscle tissue (Malm, 2001) with sustained increases in leukocyte, neutrophil, and TNF-alpha counts (Gonçalves et al., 2020; Liao et al., 2020).

Previously, preclinical studies with mice identified sustained inflammatory response for at least 24 h, tissue damage presence and multiple organ dysfunction related to practice of a type of exhaustive interval exercise named “*Repetitive bouts of exhaustive running*” (RBER) (Liao et al., 2020). Additionally, data from a study conducted with adult athletes on the impact of other models of interval physical exercise, indicate that “High-intensity training” (HIT) or “High-volume training” (HVT) promote concentrations of total leukocytes and neutrophils significantly higher after its completion with shorter rest intervals (Mathes et al., 2017). In this way, it is possible to conjecture that intensive interval physical exercise promotes inflammatory responses that are dependent on the rest periods between stimuli, thus implying a similar relationship in recovery periods between one session and another.

### Sport specificity and leukocyte morphology

Our results showed that the type of sport (combat vs court) did not have a significant effect on leukocyte morphology. This can be justified because both combat and court sports have intermittent characteristics, as well as the level of physical activity and sports level of the participants in the present study were similar regardless of the type of sport they practice. These factors may have contributed to the type of sports practiced not being significant for the findings of the present study.

Previously, when comparing responses from different protocols of high intensity interval exercise in adult athletes (age 22.1 ± 2.5 years) specialized in endurance and speed tests, no significant differences on interleukin-6 levels were observed after different types of exercise regardless of athlete specialty (endurance vs speed) (Cipryan et al., 2017). Although athlete specialties had distinct characteristics, the findings of the aforementioned study (Cipryan et al., 2017) can be justified by the fact that the sample was composed of competitors at national and/or elite levels, with high physical fitness regardless of specialty (endurance vs speed).

It is known, that in adults the level of fitness and sports competitive level (Almeida-Neto et al., 2023a; Mueller et al., 2001) apparently are more determining for effects of physical exercise on leukocyte morphology than specificity of sport itself, as suggested by curve model in “S”, it is necessary to consider intensity of exercise and fitness level to understand immune responses after exercise (Malm, 2006). Therefore, according to the findings of the present study, it is possible to suggest that curve model in “S” can be applied to adolescent athletes, however, BM rate must be considered (Almeida-Neto et al., 2023b; Malm, 2006).

### Practical applications

The findings of this study suggest that the BM rate assessed through skeletal age should be considered when monitoring leukocyte morphology after physical exercise performed by adolescent athletes. Additionally, they indicate that 2 h after the RSE, adolescent athletes are not yet fully recovered for new sessions and/or additional high-intensity physical exercise stimuli, recommending caution when prescribing multiple exercise sessions within a single day.

Considering that previous studies have found that, in adult athletes, the recovery of the immune system after a session of strenuous anaerobic or aerobic physical exercise was only complete 24 h after the sessions (Peake et al., 2017; Simpson et al., 2015; Wahl et al., 2020; Gonçalves et al., 2020), it is likely that, following the execution of RSE, the immune system is fully restored only 24 h after the stimulus. Based on this conjecture, we recommend that the minimum interval between RSE sessions in adolescent athletes, at different stages of biological maturation, should be at least 24 h.

Leukocyte morphology analysis emerges as a cost-effective tool for assessing immune system recovery following exhaustive exercise sessions, being comparatively more feasible than techniques such as flow cytometry. A total leukocyte count test, performed automatically by biomedical equipment or by a healthcare professional trained in hematological analyses using an optical microscope, can determine the neutrophil-to-lymphocyte ratio to evaluate inflammation levels and observe the dynamics of granulocytes and agranulocytes in athletes. Furthermore, the data provided in this study serve as reference values, particularly for pediatric athletes.

### Limitations and suggestions for new studies

Despite the relevant findings, the present study brings among some limitations: (i) We analyzed only male athletes; thus, caution is needed when trying to extrapolate the findings of the present study to female athletes and or non-athletes. (ii) We analyzed a cut of only 2 h after RSE, remaining hidden the behavior of leukocyte morphology in moments following this period. (iii) We did not analyze lymphocyte cell profile and or immune function, thus, findings of the present study cannot be generalized to comprehensive understanding of the effects of RSE on immune system functions. We suggest that future studies explore such limitations trying to fill these gaps in most appropriate way possible.

## Conclusion

It is concluded that the influence of repeated sprint exercise (RSE) on leukocyte morphology in adolescent athletes is dependent on the rate of biological maturation (BM). Individuals with an accelerated BM rate are less susceptible to systemic inflammation, as analyzed through the neutrophil-to-lymphocyte ratio and leukocyte level behavior, compared to their counterparts with a slower BM rate. Furthermore, up to 2 h after the RSE, adolescent athletes, regardless of their BM rate, are not yet fully recovered for new RSE sessions. It is also concluded that the type of sport practiced by adolescent athletes (combat or court sports) does not significantly affect the impact of RSE on leukocyte morphology, regardless of the BM rate.

## Data Availability

The original contributions presented in the study are included in the article/Supplementary Material, further inquiries can be directed to the corresponding author.
