# Supplementary material for: Influence of repeated sprint exercise on leukocyte morphology in adolescent athletes at different biological maturation rates
Source: Front Physiol. 2025 Mar 5;16:1480776. doi: 10.3389/fphys.2025.1480776 (PMC11919890; doi:10.3389/fphys.2025.1480776)
Supplement: Supplementary file 1 [file Table1.docx]

**Supplemental Table S-1**

**Table S-1.** General linear model to make comparisons considering the different rates of skeletal maturation (synchronized and accelerated) and the effect of time (before, after and 2h post).

| **Variable** |  | **Condition (Rate of skeletal maturation)** | **Before** | **After** | **2h Post** | **General Linear Model** | | | |
| --- | --- | --- | --- | --- | --- | --- | --- | --- | --- |
|  |  |  |  |  |  | Model | Condition | Time | P-Value |
|  |  |  |  |  |  | *Effect Size (**η^2^p \| Power)* | | |  |
| Leukocytes | g/L | Accelerated | 6.4 ± 1.5 | 8.5 ± 1.6^a^ | 7.5 ± 1.3^b^ | 0.42 \| 0.99* | 0.10 \| 0.90⸸ | 0.36 \| 0.98† | **<0.001** |
|  | g/L | Synchronized | 6.7 ± 1.2 | 10.4 ± 2.7‡^a^ | 8.5 ± 1.3‡^b^ |  |  |  |  |
| Lymphocytes | g/L | Accelerated | 2.3 ± 0.8 | 3.8 ± 1.0 ^c^ | 2.2 ± 0.5 | 0.51 \| 0.99* | 0.01 \| 0.15 | 0.50 \| 0.99† | **<0.001** |
|  | g/L | Synchronized | 2.4 ± 0.3 | 4.4 ± 1.6^c^ | 2.2 ± 0.6 |  |  |  |  |
|  | % | Accelerated | 35.4 ± 6.0 | 43.5 ± 7.5^c^ | 30.5 ± 8.5 | 0.31 \| 0.98* | 0.00 \| 0.00 | 0.29 \| 0.98† | **<0.001** |
|  | % | Synchronized | 37.4 ± 8.6 | 40.8 ± 8.01^c^ | 28.2 ± 11.1 |  |  |  |  |
| Monocytes | g/L | Accelerated | 0.3 ± 0.1 | 0.3 ± 0.1 | 0.3 ± 0.1 | 0.16 \| 0.90 | 0.06 \| 0.59 | 0.08 \| 0.82 | 0.4 |
|  | g/L | Synchronized | 0.3 ± 0.1 | 0.4 ± 0.3 | 0.3 ± 0.1 |  |  |  |  |
|  | % | Accelerated | 3.7 ± 1.5 | 3.6 ± 1.6 | 4.1 ± 0.6 | 0.05 \| 0.59 | 0.02 \| 0.15 | 0.00 \| 0.00 | 0.6 |
|  | % | Synchronized | 4.5 ± 0.6 | 4.3 ± 2.0 | 3.9 ± 1.8 |  |  |  |  |
| Segmented Neutrophils | g/L | Accelerated | 3.7 ± 0.7 | 3.8 ± 1.7 | 3.4 ± 1.9 | 0.27 \| 0.90* | 0.10 \| 0.90⸸ | 0.16 \| 0.90† | **0.03** |
|  | g/L | Synchronized | 3.3 ± 1.6 | 5.4 ± 1.4‡^a^ | 5.6 ± 1.4‡^b^ |  |  |  |  |
|  | % | Accelerated | 57.9 ± 6.1 | 50.0 ± 7.8 | 64.5 ± 8.2^b^ | 0.45 \| 0.99* | 0.00 \| 0.00 | 0.43 \|0.99† | **<0.001** |
|  | % | Synchronized | 54.4 ± 8.5 | 53.1 ± 7.0 | 68.3 ± 6.9^b^ |  |  |  |  |
| Rod Neutrophils | g/L | Accelerated | 0.1 ± 0.0 | 0.0 ± 0.1 | 0.0 ± 0.0 | 0.04 \| 0.49 | 0.02 \| 0.15 | 0.01 \| 0.15 | 0.9 |
|  | g/L | Synchronized | 0.0 ± 0.0 | 0.1 ± 0.2 | 0.1 ± 0.0 |  |  |  |  |
|  | % | Accelerated | 57.9 ± 6.1 | 50.0 ± 7.8 | 64.5 ± 8.2 | 0.01 \| 0.15 | 0.18 \| 0.90 | 0.00 \| 0.00 | 0.9 |
|  | % | Synchronized | 54.4 ± 8.5 | 53.1 ± 7.0 | 68.3 ± 6.9 |  |  |  |  |
| NLR | g/L | Accelerated | 1.7 ± 0.4 | 1.1 ± 0.6 | 2.1 ± 1.2^b^ | 0.34 \| 0.98* | 0.02 \| 0.15 | 0.30 \| 0.98† | **<0.001** |
|  | g/L | Synchronized | 1.4 ± 0.8 | 1.4 ± 0.8 | 2.8 ± 1.2^b^ |  |  |  |  |

NLR: Total neutrophils/lymphocytes ratio. g/L: Gram per liter. %: Percentage. *: Statistically significant model. †: Significant time factor (p<0.05). ⸸: Significant condition (p<0.05). ‡: Higher than the Accelerated group [Post hoc Bonferroni (p<0.05)]. ^a^: Higher than the Before moment [Post hoc Bonferroni (p<0.05)]. ^b^: Superior to the Before and After moments [Post hoc Bonferroni (p<0.05)]. ^c^: Higher than Before & 2h post moments [Post hoc Bonferroni (p<0.05)].
